# Cognitive dysfunction in Nigerian women with epilepsy on carbamazepine and levetiracetam monotherapy

**DOI:** 10.1002/brb3.2038

**Published:** 2021-03-05

**Authors:** Luqman Ogunjimi, Joseph Yaria, Akintomiwa Makanjuola, Akinyinka Alabi, Bamidele Osalusi, David Oboh, Mojisola Olusola‐Bello, Adeyinka Aderinola, Adesola Ogunniyi

**Affiliations:** ^1^ Department of Pharmacology and Therapeutics Obafemi Awolowo College of Health Sciences Olabisi Onabanjo University Sagamu Nigeria; ^2^ Department of Medicine University College Hospital Ibadan Nigeria; ^3^ Department of Medicine Obafemi Awolowo College of Health Sciences Olabisi Onabanjo University Sagamu Nigeria; ^4^ Department of Radiology University College Hospital Ibadan Nigeria; ^5^ Radiology Department Obafemi Awolowo College of Health Sciences Olabisi Onabanjo University Sagamu Nigeria

**Keywords:** cognition, epilepsy, women

## Abstract

**Background:**

This study aims to identify the determinants of cognitive dysfunction and compare the effect of CPZ and LTC on cognition in WWE.

**Methods:**

An observational study involving 87 consenting adult WWE aged between 16 and 40 years on LTC or CZP monotherapy. At enrollment, an interviewer‐based questionnaire was used to obtain demographic and clinical information from participants. The diagnosis of epilepsy was mainly clinical and supported by electroencephalographic (EEG) features and classified based on recommendation by the 2017 International League Against Epilepsy (ILAE). Zung Self‐Reporting Depression Scale (ZSRDS) was used to assess the mood of participants. The Community Screening Interview for Dementia (CSID) was used to assess various cognition domains. The National Hospital Seizure Severity Scale (NHS‐3) was used to assess disease severity.

**Results:**

There were statistical differences between the CZP and LTC groups in all domains of cognition assessed except for orientation. The total CSID scores of the LTC group were 59.2 (4.9) as opposed to CZP group, 57.2 (5.0); *p*: .005. Those with focal onset seizures had lower median total CSID score (58; IQR: 54–62) when compared to those with generalized onset seizures (62; IQR: 58–62), *p*: .012. There was a significant correlation between ZSRD score and NHS‐3 score; rho: 0.30, *p*: .007. Bivariate analysis shows statistically significant correlation between total CSID score and ZSRDS (rho: −0.65), BMI (rho: 0.22), and NHSS‐3 score (rho: −0.36), respectively. However, the effect of AED on CSID scores was lost after multivariate quantile regression with only ZSRDS retaining significance.

**Conclusion:**

Depression, seizure severity, type and structural etiology were associated with cognitive impairment among WWE. However, on regression model, only depression was statistically significant. The presence of more risks for cognitive impairment in the CZP group limits possible conclusion of LTC superiority.

## Graphical abstract

We set out to identify the determinant of cognitive dysfunction and compare the effect of CPZ and LTC on cognition in women with epilepsy. Depression, seizure severity, type, and structural etiology were associated with cognitive impairment among WWE.However, on regression model, only depression was statistically significant. The presence of more risks for cognitive impairment in the CZP group limits possible conclusion of LTC superiority.

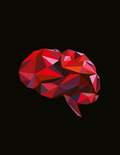

## 1 INTRODUCTION

Cognitive dysfunction has been described in people with epilepsy when compared to controls (Mwangala et al., 2018; Sen et al., 2018; Sunmonu et al., 2009). This has been linked to several factors including epilepsy and the cumulative effect from so AEDs (Ogunrin et al., 2000; Sunmonu et al., 2009). The pathogenic mechanism involved in cognitive impairment includes oxidant stress, apoptosis, excitotoxicity, neuronal loss, calcium toxicity, ischemia, and depletion of choline acetyltransferase (Vishnoi et al., 2016; H. Yang, Chan, et al., 2018). Risk factors for cognitive impairment in people with epilepsy include pharmaco‐resistance, duration of epilepsy, age of onset, severity of epileptic activity, ictal and interictal epileptiform pattern, psychosocial factors, and underlying acquired and structural lesion (Loughman et al., 2017; Taylor et al., 2011; H.‐L. Yang, Chan, et al., 2018). Recent findings show that older persons with epilepsy are more likely to suffer from cognitive dysfunction and that there might be an important bidirectional relationship between epilepsy and dementia (Ogunrin et al., 2000; Sen et al., 2018).

The effect of AEDs on cognition has been elucidated (Helmstaedter & Witt, 2008; Sen et al., 2018; Sunmonu et al., 2009). It has been postulated that the effect of older enzyme inducing agent and newer nonenzyme inducing antiepileptic agents varies on quality of life and cognitive function (Klitgaard & Pitkänen, 2003; Svalheim et al., 2009). Studies have shown that problems of enzyme induction and pharmacokinetic interactions with older AEDs are some of the issues favoring newer AEDs over choice of CZP. Several newer‐generation AEDs have undergone comparative trials demonstrating efficacy equal to and tolerability at least equal to or better than older AEDs as first‐line therapy (Abou‐Khalil, 2016).

CPZ, one of the most common AEDs used in sub‐Saharan Africa (SSA), has been shown to have the best balance of efficacy and tolerability when compared to phenytoin, phenobarbital, and primidone. (Abou‐Khalil, 2016; Mattson et al., 1985). LTC is the most common of the newer AEDs used in SSA has been reported to have excellent pharmacokinetic neuroprotective, antiepileptogenic, and cognitive enhancing properties. (Chakravarthi et al., 2015; Fukuyama et al., 2012). LTC inhibits IL‐1b inflammatory responses and reduced reactive gliosis in the hippocampus and piriform cortex in a rat model of epilepsy suggesting it could be an important agent in the prevention of epileptogenesis (Kim et al., 2016). Furthermore, in clinical studies, LTC was associated with improved cognitive or neural functional outcomes after intracerebral hemorrhage. (Taylor et al., 2011) It is rather surprising that fewer studies have compared the differential effect of older enzyme inducing AEDs (CPZ) with newer nonenzyme‐inducing drug (LTC) with respect to cognition. This study aims to identify the determinant of cognitive dysfunction and compare the effect of CPZ and LTC on cognition in WWE.

## 2 METHODS

An observational study involving 87 WWE on treatment attending the Neurology Out‐Patient Department of the University College Hospital (UCH), Ibadan. Using the clinic record as sampling frame, cases who met the inclusion criteria were randomly selected from pool of WWE into the study with 46 (52.9%) on CZP and 41 (47.1%) on LTC. Sample size calculation for comparison of means was used with mean total CSID scores in epilepsy obtained from previous local study, and expected difference in CSID score of three was postulated (Sunmonu et al., 2009). Inclusion criteria for the study were consenting adult WWE aged between 16 and 40 years on LTC or CZP monotherapy for at least 6 months. Patients on multiple AEDs, prior or current use of alternative medicine, those with premorbid cognitive impairment, retroviral infection, previously or recently diagnosed diabetes, and those on hormonal replacement therapy were excluded. This study was carried out after due ethical clearance was obtained from the Joint Institution Review Committee (IRC) of the University College Hospital and the College of Medicine, University of Ibadan.

At enrollment, an interviewer‐based questionnaire was used to obtain demographic and clinical information from participants. Epilepsy‐related variables of interest included age of onset, seizure semiology, previous AED, seizure frequency, and postictal status among other details. The diagnosis of epilepsy was mainly clinical and supported by electroencephalographic (EEG) features. Epilepsy was classified based on recommendation by the 2017 International League against Epilepsy (ILAE) (Scheffer et al., 2017). Seizure frequency is defined as the average number of seizures per month over the last 3 months based on hospital records and patient reports. Seizure control was defined using 2017 ILAE criteria (Brodie et al., 2018). ZSRDS was used to assess the mood of participants (Dunstan et al., 2017; Romera et al., 2008). The CSID was used to assess various cognition domains (Hall et al., 2000). The CSID is a culturally acceptable, paper and pen‐based global cognitive function screening instrument developed by Ibadan‐Indianapolis dementia project which assesses language, memory, calculation, orientation, and attention subdomain. It has been used successfully assess cognitive function in patient with epilepsy, heart failure, asthma, and chronic liver disease (Adebayo et al., 2016; Hall et al., 2000; Sunmonu et al., 2009, 2019). The National Hospital Seizure Severity Scale (NHS‐3), which contains seven seizure‐related factors and generates a score from 1 to 27, was used to assess disease severity (O’Donoghue et al., 1996). All the instruments mentioned above (ZSRDS, CSID, NHS‐3) were previously pretested and validated in our community (Hall et al., 2000; Imam et al., 2005; Ogunrin et al., 2000; Okubadejo et al., 2007; Yaria et al., 2019). Using the international 10–20 electrode placement, electroencephalography was carried out on all the participants with a Phoenix digital 16‐channel electroencephalography machine. These recordings were done by a trained electrophysiologist with each recording taken at least 30 min including activation procedures (hyperventilation and photic stimulation), and reports were interpreted by a neurologist. Epileptiform abnormalities were defined as sharp waves with accompanying slow waves complexes, or spikes with accompanying slow‐wave discharges that are distinct from the normal background activity. (Maganti and Rutecki, 2013).

Statistical analysis was done using Stata version 12 by StataCorp LLC. Outcome variables of interest for this study were cognitive scores. Bivariate analysis was carried out using the independent Student *t* test, and Pearson chi‐square test while Mann–Whitney *U* test and Spearman correlation were used for nonparametric variables. Due to the nonparametric distribution of variables and the suspicion of small dataset, bootstrap resampling methods based on observations (B = 1,000 bootstrapped samples) were carried out to evaluate the effect of various factors on CSID scores. Variables that attained statistical significance on bivariate analysis were imputed into a bootstrapped quantile regression model. Quantile regression was chosen since it is more robust with outliers, as the distribution of cognitive scores was skewed. The level of statistical significance was set at *p*‐value of < .05.

## 3 RESULTS

### 3.1 Socio‐demographic and Clinical Characteristics of Participants

Eighty‐seven patients were randomly sampled from pool of WWE into the study with 46 (52.9%) on CZP and 41 (47.1%) on LTC. As shown in Table 1, there was no difference in mean age of participants between groups, *p*:.151. With respect to other socio‐demographic variables considered, there was no statistically significant difference between the CZP and LTC group. Despite similar BMI, the CZP group had a higher waist circumference: 83.4 (10.3) cm and hip circumference: 88.2 (12.8) cm, when compared with the LTC who had a waist circumference of 78.7 (11.4) cm and hip circumference of 82.6 (11.9) cm, *p*:.037, respectively. Very few of the recruited participants were hypertensive, 2 (4.4%) of the CZP group and 6 (14.6%) of the LTC group, *p*:.141. The CZP group however had a higher Zung score when compared to the LTC group, 0.048. Also, there was a significant correlation between Zung score and NHS‐3 score; rho: 0.30, *p*:.007.

**TABLE 1:** Socio‐demographic and clinical characteristics of participants

	Carbamazepine	Levetiracetam	*p*‐value
Age, mean (*SD*)	30.3 (8.1)	27.9 (7.3)	.207
Education, *N* (%)			
Primary	18 (39.1)	8 (19.5)	.137
Secondary	15 (32.6)	15 (36.6)	
Tertiary	12 (26.1)	14 (34.1)	
Postgraduate	1 (2.2)	4 (9.8)	
Marital status, *N* (%)			
Single	32 (69.6)	27 (65.8)	.819
Married	14 (30.4)	14 (34.2)	
Alcohol, *N* (%)	1 (2.2)	2 (5.0)	.595
BMI, Mean (*SD*)	24.3 (4.4)	24.3 (4.2)	.996
Waist circumference, mean (*SD*)	83.4 (10.3)	78.7 (11.4)	.037*
HIP circumference, mean (*SD*)	88.2 (12.8)	82.6 (11.9)	.037*
ZSRDS, median (IQR)	32 (28–36)	28 (25.5–33)	.048*
Hypertensive, *N* (%)	2 (4.4)	6 (14.6)	.141

Abbreviation: ZSRDS, Zung Self‐Reporting Depression Scale.

* Statistically significant.

### 3.2 Epilepsy‐related characteristics of participants

There was no significant difference in most epilepsy‐related variables as shown in Table 2. However, 33 (71.7%) of the CZP group were deemed to have a structural lesion as seizure etiology as opposed to 19 (46.3%) from the LTC, *p*‐value:.022. Participants in the CZP group had a higher mean NHSS‐3 score when compared to those in the LTC group, *p*:.012.

**TABLE 2:** Epilepsy‐related characteristics of participants

	Carbamazepine *N* = 46	Levetiracetam *N* = 41	*p*‐value
Duration in years, median (IQR)	7.5 (1.5–14)	5 (3–10)	.675
Last Seizure Episode, *N* (%)			
1–2 years	26 (56.6)	26 (63.5)	.193
2–5 years	14 (30.4)	14 (34.1)	
>5 years	6 (13.0)	1 (2.4)	
Classification			
Focal onset	24 (52.2)	21 (51.2)	.550
Generalized onset	22 (47.8)	20 (48.8)	
Timing, *N* (%)			
Nocturnal	4 (8.7)	5 (12.2)	.729
Anytime	42 (91.3)	36 (87.8)	
NHSS−3 score, mean (*SD*)	16.2 (6.5)	12.6 (6.4)	.012*
Head trauma, *N* (%)	2 (4.4)	4 (9.8)	.415
Regular medication, *N* (%)	41 (89.2)	31 (75.6)	.096

Abbreviation: NHSS‐3, National Hospital Seizure Severity Scale Score.

* Statistically significant.

### 3.3 Medication effect on Cognition

There were statistical differences between the CZP and LTC groups in all domains of cognition assessed except for orientation. WWE on LTC had better overall language, memory, attention, and total CSID scores though marginal. The total CSID scores of the LTC group were 59.2 (4.9) as opposed to CZP group, 57.2 (5.0); *p*:.005. Concerning language subdomain, the LTC group had higher average comprehension and fluency scores, *p*:.020 &.014, respectively. Also, in the memory domain, significant difference was attained for registration and semantic memory with the LTC group also having higher scores, *p*:.006 & .001, respectively, as shown in Table 3.

**TABLE 3:** Cognitive screening instrument for dementia scores of participants

	Carbamazepine	Levetiracetam	*p*‐value
Language, mean (*SD*)	22.0 (1.7)	22.6 (1.6)	.005*
Expression			
Naming	6.9 (0.3)	6.8 (0.5)	.535
Definition	4.8 (0.4)	4.9 (0.2)	.069
Fluency	4.6 (0.6)	4.8 (0.5)	.020*
Total	17.4 (1.2)	17.8 (0.9)	.011*
Comprehension	4.6 (0.6)	4.8 (0.6)	.014*
Memory, Mean (*SD*)	18.3 (2.7)	19.5 (2.5)	.006*
Registration	3.4 (0.7)	3.8 (0.5)	.006*
Immediate recall	5.5 (0.9)	5.5 (0.9)	.948
Delayed recall	1.8 (0.4)	1.8 (0.4)	.801
Semantic	7.8 (1.3)	8.4 (1.4)	.001*
Attention and calculation, Mean (*SD*)	7.2 (1.0)	7.5 (1.1)	.014*
Orientation, mean (*SD*)	9.9 (0.3)	9.9 (0.3)	.935
Total CSID, Mean (*SD*)	57.2 (5.0)	59.2 (4.7)	.005*

* Statistically significant

### 3.4 Relationship between Cognition and other clinical parameters

Bivariate analysis shows statistically significant correlation between total CSID score and Zung depression score (rho: −0.65), BMI (rho: 0.22), and NHSS‐3 score (rho: −0.36), respectively (Table 4). There was however no significant difference in median total CSID score between those regular on medication and those who were not (p: 0.309). However, those with focal onset seizures had lower median total CSID score (58; IQR: 54–62) when compared to those with generalized onset seizures (62; IQR: 58–62), *p*:.012.

**TABLE 4:** Relationship between cognition and clinical parameters

	Rho	*p*‐value
Age	−0.02	.857
Waist circumference	0.04	.722
Hip circumference	0.11	.313
BMI	0.22	.040*
ZSRDS	−0.65	<.001*
NHSS−3 score	−0.36	.001*
Duration of epilepsy	−0.21	.055

Abbreviations: NHSS‐3, National Hospital Seizure Severity Scale Score; ZSRDS, Zung Self‐Reporting Depression Scale; BMI, body mass index.

* Statistically significant.

### 3.5 Quantile regression model

Table 5 shows the results from the quantile regression using the total CSID as dependent variable. An increase in Zung score was significant the lower conditional quartile in the 25th and 50th quartiles with greater impact on cognition of CSID scores. The AED type, seizure severity, etiology, and regularity of medication used did not attain any statistical significance.

**TABLE 5:** Quantile regression for total cognitive score

	25th Quartile	50th Quartile	75th Quartile
Education	0.74 (0.406)	0.43 (0.435)	−0.11 (0.824)
Medication class	−0.14 (0.711)	0.24 (0.571)	0.27 (0.517)
Waist circumference	0.02 (0.794)	0.01 (0.731)	−0.005 (0.984)
BMI	0.14 (0.335)	0.16 (0.260)	0.02 (0.830)
ZSRDS	−0.54 (<0.001)^*^	−0.40 (0.003)^*^	−0.19 (0.133)
Seizure severity	−0.06 (0.630)	−0.08 (0.388)	−0.04 (0.587)
Duration of epilepsy	0.27 (0.701)	0.02 (0.947)	0.23 (0.622)
Seizure etiology	0.36 (0.158)	0.06 (0.783)	0.03 (0.909)
Regular on medication	1.60 (0.529)	0.11 (0.932)	−0.43 (0.629)

Note

R2: 0.353, 0.292, 0.126.

Abbreviations: NHSS‐3, National Hospital Seizure Severity Scale Score; ZSRDS, Zung Self‐Reporting Depression Scale; BMI, body mass index.

* Statistically significant.

### 3.6 Differential correlates of cognitive status based on medication class

Table 6 shows differential correlates of cognitive status in both CZP and LTC group of WWE. Bivariate analysis shows statistically significant correlation between total CSID score and Zung depression score in both groups (rho: −0.88, *p*‐value < .001 in the CZP group, rho −0.39, *p*‐value .013 in the LTC group). In the LTC group, there is a correlation between ZSRD score (Rho −0.39, *p*‐value .013), BMI (Rho 0.49, *p*‐value .001), and waist circumference (Rho 0.32, *p*‐value .042).

**TABLE 6:** Differential correlates of cognitive status based on medication class

	CZP		LTC	
	Rho	*p*‐value	Rho	*p*‐value
Age	−0.14	.337	0.23	.142
Waist circumference	−0.08	.586	0.32	.042^*^
HIP circumference	0.12	.425	0.19	.224
BMI	−0.02	.904	0.49	.001^*^
ZSRDS	−0.88	<.001^*^	−0.39	.013^*^
NIHSS−3 SCORE	0.05	.764	−0.53	<.001^*^
Duration of epilepsy	0.29	.049	−0.28	.079

Abbreviations: NHSS‐3, National Hospital Seizure Severity Scale Score; ZSRDS, Zung Self‐Reporting Depression Scale; BMI, body mass index.

* Statistically significant.

## 4 DISCUSSION

To the best of our knowledge, this is first study comparing the cognitive effect of LTC and CZP in WWE in Nigeria and sub‐Saharan Africa by extension. First, while patients on LTC performed better than those on CZP across all cognitive domains except orientation, the presence of more risks for cognitive impairment in the CZP group limits such conclusion. In this study, depression, seizure severity, type, and structural etiology were associated with cognitive impairment among WWE. However, on regression model, only depression and not medication class, etiology, and seizure severity attained a statistically significant level. A previous study by Adebayo et.al reported higher frequency of depression and poorer quality of life among of WWE compared to controls (Adebayo et al., 2014). The correlation between total CSID score and Zung depression score supports the finding from previous description of cognitive deficit across all domains which has been long described in depression. The implicated mechanism includes the dysregulation of emotion in depression, inhibitory process and deficit in working memory, ruminative responses to negative mood sates and negative life events, and the inability to use positive and rewarding stimuli to regulate negative mood (Chakrabarty et al., 2016; Coppola et al., 2019). In line with previous reports, we identified depression, seizure severity, seizure type, and structural etiology as major associates of cognitive status from this study. (Chakrabarty et al., 2016; Mwangala et al., 2018; van Rijckevorsel, 2006) Considering the type of seizures, those with focal onset seizures had lower median total CSID score when compared to those with generalized onset seizures indicating possibility of poorer cognitive function in those with focal seizures. Previous studies have also described significant cognitive decline in patient with focal epilepsy compare to generalized epilepsy especially memory domain. However, the trajectory of cognition depends on age of onset, etiology severity, and semiology (Liguori et al., 2019; van Rijckevorsel, 2006; Sen et al., 2018). In a study by Arinzechi and colleagues aimed at assessing cognitive performance in epileptic performance in rural southeastern Nigeria, it was demonstrated patient with high seizure frequency had higher prevalence and risk of memory impairment but not in other neurocognitive domain when compared with those with low seizure frequency. (Arinzechi et al., 2019).

In another study aimed at identifying distinct autobiographic memory dysfunction in patients with epilepsy focusing on early versus late disease onset, Rayner and colleagues identified different reason for reduced autobiographic memory in patients with early‐ and late‐onset epilepsy. These include young age at onset, frequent seizures and reduced working memory in childhood onset seizures while it was linked to structural anomaly on imaging and depression in those with adult onset seizures. (Rayner et al., 2016) Structural lesions carry an additive risk of cognitive dysfunction with cumulative effects on cognitive network which might explain while cases on CZP (with significant structural etiology) had poorer cognitive function compared to those on LTC.

A number of studies have reported worsening of cognition with CZP use and improvement with LTC use (Eddy et al., 2011; Huang et al., 2015). Previous study showed LTC seemed to improve reaction time, tapping rate, and memory when compared with other AEDs following a detailed neuropsychological assessment (Magalhães et al., 2015; Taylor et al., 2011). LTC action include enhancement of executive function, working memory, fluid intelligence, and verbal fluency psychomotor speed with fewer untoward neurophysiological and neuropsychological effect compare with CZP. (Eddy et al., 2011; Magalhães et al., 2015; Meador et al., 2007; Wu et al., 2009) LTC activities might be related to its positive activities at the synaptic vesicles, excellent pharmacokinetic property, anti‐inflammatory, and antiepileptogenic property (Abou‐Khalil, 2016; Klitgaard & Pitkänen, 2003; Löscher & Brandt, 2010). Kim et al. showed LTC inhibited IL‐1b inflammatory responses and reduced reactive gliosis in the hippocampus and piriform cortex in a rat model of epilepsy suggesting it could be an important agent in the prevention of epileptogenesis. (Kim et al., 2010) This neuro‐protective role especially on the hippocampus is vital in enhancing memory and cognitive performance.

On the contrary, CZP has potential to lead to significant difficulties with regard to motor speed, reaction time, and memory performance on attention demanding tasks (Eddy et al., 2011; Meador et al., 2007). However, AEDs were not an independent predictor of cognition in this study; as such, it will be difficult to ascribe the cognitive changes from this study to medication effect.

### 4.1 Limitation

The study design is cross‐sectional and not longitudinal nor prospective randomized control trial (RCT); as such, it is difficult to ascribe the cognitive changes to medication. There is need for a prospective RCT or longitudinal study to ascertain further evaluates the role of medication with regard to cognition. Again while depression, seizure severity, and structural etiology are major cofounders to ascribing cognitive changes to AEDs use in this study, these factors were controlled for on the regression model which shows only depression and not medication class, etiology, and seizure severity attained a statistically significant level.

Apart from the fact that study participants were only females, the study is also limited by the fact that we did not include other relatively commonly used AEDs but focused on comparison between a relatively newer AED (LTC) and a more commonly prescribed older AED (CZP) in our environment. Nevertheless, while proposing a longitudinal or prospective RCT to further evaluate the role of LTC/CZP monotherapy, we have been able to identify depression as a major determinant of cognitive status in WWE.

## 5 CONCLUSION

Depression, seizure severity, type, and structural etiology were associated with cognitive impairment among WWE. However, on regression model, only depression was statistically significant level. Furthermore, medication was not an independent predictor of cognition in this study; as such, it will be difficult to ascribe the cognitive changes from this study to medication effect. Though LTC performs better than those on CZP across all cognitive domains except orientation, the presence of more risks for cognitive impairment in the CZP group limits such conclusion. Physicians and healthcare providers should pay attention not only to cognitive dysfunction and its determinants but also the choice of AEDs with the aim of improving quality of life among WWE.

## CONFLICT OF INTEREST

The authors declare that there is no conflict of interest.

## AUTHORS’ CONTRIBUTION

Luqman, Joseph, and Adesola conceived the idea of the study. Luqman, Joseph, Akinyinka, Moji, David, Adeyinka, Akintomiwa, Bamidele, and Adesola involved in the study design, data collection, analysis, and interpretation and made significant intellectual contribution manuscript development. Joseph and Luqman handled statistical analysis. Moji and David involved in neuro‐imaging acquisition, interpretation, and adjudication.

### Peer Review

The peer review history for this article is available at https://publons.com/publon/10.1002/brb3.2038.

## Data Availability

The data will be made available upon tangible request to principal investigator, Luqman. Luqman and Joseph have the final responsibility for the decision with regard to full access to the study data and manuscript submission.
